# Factors Affecting Green Purchase Intention: A Perspective of Ethical Decision Making

**DOI:** 10.3390/ijerph191811151

**Published:** 2022-09-06

**Authors:** Ziyuan Tian, Xixiang Sun, Jianguo Wang, Weihuan Su, Gen Li

**Affiliations:** School of Management, Wuhan University of Technology, Wuhan 430070, China

**Keywords:** moral intensity, moral judgment, perceived quality, perceived price, products’ green degree, green purchase intention

## Abstract

Environmental protection issues are closely related to moral factors, but little research has explored the factors affecting green purchase intention from the aspect of morality. Based on a perspective of ethical decision making, this study investigates the impacts of perceived quality and perceived price on moral intensity and moral judgment, through the moderation of products’ green degree, as well as the impacts of moral intensity and moral judgment on green purchase intention. Research data was collected through a questionnaire survey of 368 consumers in China, and analyzed using a hierarchical linear model. The empirical results reveal that moral intensity and moral judgment significantly promoted green purchase intention. Perceived quality and perceived price both positively affected moral intensity and moral judgment. Products’ green degree positively moderated the relationship between perceived price and moral judgment as well as the relationship between perceived quality and moral judgment. This study provides a new insight into promoting green purchase intention, and the findings may also assist marketers in developing future tactics to increase consumers’ green purchase intention.

## 1. Introduction

Rapid economic expansion and technological innovation have improved humans’ lives, but they also cause various environmental hazards, including atmospheric contamination, water pollution, and destruction of biodiversity. These challenges directly affect the long-term viability of society and the environment [1]. According to correlative investigation, personal and household consumption has been responsible for 30–40% of the degradation of the ecological environment [2]. To some degree, governments’ environmental protection measures and businesses’ positive responses can accelerate the progress of green consumption modes. However, consumers’ lifestyles and consumption patterns are the end point of green consumption and are the primary focus of efforts to increase green consumption habits. As a result, converting consumption patterns into green consumption with fewer adverse effects on the environment is essential for achieving peaceful cohabitation of humans and nature [3]. Populations have begun to attach greater significance to environmental preservation and have tended steadily to expand their usage of environmentally friendly items. Especially throughout the COVID-19 epidemic, societal beliefs and behavior patterns shifted towards a more secure, healthier, and more environmentally friendly orientation [4,5]. Accordingly, the manufacture and marketing of green products have garnered attention from enterprises. Business leaders and professionals have been looking for methods to persuade consumers to buy environmentally friendly items [6].

Green purchase intention has been described as “the consumers’ intention to purchase and pay for green products” [7]. Green purchase intention is regarded as a sort of pro-environmental consumerism, because green products assist the environment by conserving resources or energy while minimizing or removing pollutants, hazardous waste, and the usage of dangerous chemicals, and continuing to meet consumers’ needs [8]. Various research has indicated that the desire to make green purchases is progressively growing [9,10,11]. Previous studies have determined that the antecedents determining green purchase intention essentially comprise consumer demographics, social–psychological characteristics, product attributes, and additional contextual elements. Demographic characteristics of consumers, including gender, education [12], age [13], and income [14] have been discovered to be determining factors of green purchase intention. In their investigations, Chekima et al. [12] revealed that compared to male customers, female consumers were more likely to make green purchases, and those with higher levels of education were more likely to have green purchase intention than those with lower education levels. Sun et al. [13] argued that the likelihood of making a green purchase increases with age. In terms of income, consumers with higher incomes are more inclined to buy green goods and services [14]. In addition, green purchase intention is largely determined by social–psychological factors, such as green trust [13], values [15], self-identity [16], social norms [17], and reference groups [18]. For example, Xu and Lin [17] observed that peer approbation impacted college students’ inclination to make green purchases. Some research findings suggest that product attributes such as price [19], packaging [20], and advertising [17,21] are among the most critical elements motivating people to buy green products. Finally, other contextual elements include perceived incentives [22], green communication [23], environmental responsibility [24], environmental commitment [25], and environmental consciousnesses [26]. For example, Yue et al. [24] drew a conclusion that environmental concern and greener consumption can both be enhanced by environmental responsibility.

Environmental protection issues are strongly linked to moral factors [27,28,29], but there has been insufficient research on the factors influencing green purchase intention from a moral perspective. The ethical decision-making sequence model put forward by Rest [30] considers moral awareness, moral judgment, moral intention, and moral conduct, in order. Jones [31] supplemented the model by further taking into account issue-dependent variables, such as the magnitude of behavioral outcomes. Jones [31] classified these circumstance-dependent elements as moral intensity. There have been some studies of the effects of ethical decision making on purchase intention. For instance, the study by Tan [32] proved that consumers’ purchase intention for pirated software was influenced by moral intensity and moral judgment. Martinez and Jaeger [33] emphasized the significance of moral awareness as an indispensable component of ethical decision making, and confirmed that moral emotions affected moral judgment as well as counterfeit purchase intention. Andersch et al. [34] indicated that moral judgment and moral obligation play mediating roles in the influence of negative effects on ethical purchase intention. Moreover, the effect of stage-model components on moral judgment and ethical purchase intention may be moderated by egoistic purchase motives. Although the above research addressed moral factors, there has been little discussion of how moral factors impact consumers’ green purchase intention. Since consumers’ willingness to carry out green purchase behavior largely depends on moral judgment and moral intensity [35], this study explores the factors affecting green purchase intention from a perspective of ethical decision making.

In the course of ethical decision making, decision makers are influenced by value [36], work experience [37], moral intensity [38], and many internal personal factors. Among these influences, value is one of the key elements affecting ethical decisions, because when consumers purchase goods, they form a perceived value for the commodity, thus influencing their purchase intention [39,40,41]. Consumers evaluate the final benefits that they may obtain under different situations, that is, they make a compromise between perceived benefit and perceived sacrifice [42,43], and then form a perceived value to choose whether to purchase a product or service. According to Dodds and Monroe [44], perceived benefit can be regarded as perceived quality, and perceived sacrifice as perceived price. In the interaction of these two factors in the mind of the consumer, the process of ethical decision making for green purchasing may occur. Past research has mostly focused on the direct impact of value on green purchase intention [45,46,47], few studies have investigated the intermediate process regarding the influence of perceived price and perceived quality on green purchase intention. Therefore, this study further explores the impact of consumers’ perceptions of quality and price on moral judgment and moral intensity.

In the context of big data, Liu and Yi [48] analyzed the influence of advertising and products’ green degree on pricing policies, and found that products’ degree of greenness was a significant factor affecting sales within the supply chain of green industry. The enhancement of greenness improves the natural ecology and living environment, and may bring more positive moral attitude. On the other hand, since the greenness of different manufacturers may be different, this study takes this difference into consideration and discusses the degree of greenness at a group level. In the process of research, the nested structure between group-level and individual-level data cannot be ignored [49]. Consequently, this study adopted a hierarchical linear model, using products’ green degree as a moderating variable, to investigate how consumers’ perceived quality, perceived price, moral judgment, and moral intensity impact their green purchase intention. This represents a partial attempt to address the aforementioned gaps. Through an understanding of the psychological factors of consumers, the study findings can provide strategic reference for enterprise management, thus enhancing the green purchase intention of consumers.

## 2. Literature Review and Hypothesis Development

### 2.1. Moral Intensity and Green Purchase Intention

Moral intensity describes “the extent of issue-related moral imperative in a situation”. The moral intensity of consumers is likely to vary according to the effects of different factors, so it is necessary to judge its reliability and stability based previous experience and research [31]. Moral intensity is an important antecedent when making ethical decisions [30,50].

Previous research has demonstrated that moral intensity is correlative with ethical reasoning when moral issues are identified, moral judgments are made, behavioral intentions are established, and moral behaviors occur [51,52]; at every stage, moral intensity stimulates people to use ethical reasoning. For example, Leitsch [53] examined responses of 110 students majoring in accounting and came to a conclusion that moral intensity is relevant to moral sensitivity, moral judgments, and intentions. Sweeney and Costello [54] made the argument that the first three steps in making an ethical choice are influenced by moral intensity. Barnett and Valentine [55] and Barnett [56] suggested that assessments requiring moral intensity have connections with students’ abilities to identify ethical issues, make moral judgements, and create behavioral objectives. Hong [57] found that customers’ purchase behavior towards organic and natural-hued textiles and clothing items was favorably influenced by moral intensity. Bray et al. [58] indicated that the greatest barrier to ethical purchasing is consumers’ limited understanding of the (un)ethical repercussions of their purchases. Therefore, if consumers are conscious of the outcomes and impact of their green purchase behavior, and recognize the significance of their choices, their green purchase intention will increase. Consequently, we suggest the hypothesis:

**Hypothesis** **1** **(H1).** *Moral intensity is positively correlated with green purchase intention.*

### 2.2. Moral Judgment and Green Purchase Intention

Moral judgment describes personal “prescriptive assessment of what is right or wrong” [59]. It involves assessing which actions are morally justified in response to an ethical issue [60].

Moral judgment has aroused widespread interest among academic researchers, and it is often employed in ethical decision-making models to account for moral conduct [31,61]. Previous studies have proved that personal moral judgment affects moral intention or moral behavior; for instance, Tan [32], Moores and Chang [62], and Wagner and Sanders [63] all verified that the greater a person’s moral judgment, the less likely he is to acquire pirated software. Additionally, Ha and Lennon [64] investigated consumers’ moral judgment regarding counterfeit fashion products and came to the same conclusion. Accordingly, an individual who evaluates the behavior of purchasing a counterfeit product as morally reprehensible is unlikely to buy that item. Therefore, the existing research implies that consumers’ moral judgment is influenced by the moral factors relating to purchasing certain goods or services, in addition to their own cognition, and that this affects their purchase intention.

Given the aforementioned analysis, it is reasonable to suggets that when the moral factors of a product or service are more positive, consumers’ moral judgment will be improved, and their green purchase intention will be enhanced. On the contrary, when the moral factor of the product or service is more negative, the moral judgment of consumers will be reduced, so that the green purchase intention will remain unchanged or decline. Consequently, we suggest the hypothesis:

**Hypothesis** **2** **(H2).** *Moral judgment is positively correlated with green purchase intention.*

### 2.3. Perceived Price, Moral Intensity and Moral Judgment

Price stands for the expenditure involved in every purchase transaction [65]. Zeithaml [66] defined price as “what is given up or sacrificed to acquire a product”. Jacoby and Olson divided price into objective or perceived [67]; objective price represents a product’s real price, perceived price is the consumer’s subjective view of the objective price [67]. When making purchasing selections, consumers usually make a comparison between objective price and internal reference price. Internal reference price is the consumer-perceived price range for the product classification [68]. After consumption, customers encode the price of a certain item or service in a way that makes sense to them, rather than remembering the real cost [69]. For example, although some customers may not recall the exact price of a product or service, they are more likely to recall the pricing as “expensive” or “cheap”.

Much research has shown that perceived price is a significant determinant influencing a consumer’s purchase intention. For instance, Ramadhan and Muthohar [70] revealed that customers’ intention to acquire hypermarket private-label items was significantly influenced by perceived price. The findings of Satriawan [71] indicated that Xiaomi smartphone purchase intention was favorably and strongly influenced by perceived quality and perceived price. The reference price affecting the perceived price range is formed by the consumer’s past consumption experience. Therefore, if a consumer’s perceived price for a particular product is too high in comparison with that of the homogeneous product, his purchase intention will decrease. Unless the overall benefit and value of the product is sufficient to make up for a certain price gap, consumers may feel less ethically motivated. Generally speaking, the perceived price and overall value of green products are greater than those of products that are less environmentally friendly. Therefore, this study believes that the overall benefits and value of green products can to a certain extent mitigate the gap between consumers’ internal reference prices and thereby reduce the possibility of unethical feelings.

Moreover, the moral environmental factors represented by green products can also arouse the moral identity of consumers, thereby enhancing moral intensity and moral judgment. Therefore, when consumers perceive a higher price for a green product, they naturally form a higher moral judgment evaluation of the product. Correspondingly, when consumers evaluate the perceived price of a green product as higher, the moral intensity of the green product increases. Given that analysis, this study argues that higher perceived price will promote consumers’ moral intensity and moral judgment. Consequently, we suggest the hypotheses:

**Hypothesis** **3** **(H3).** *Perceived price is positively correlated with moral intensity.*

**Hypothesis** **4** **(H4).** *Perceived price is positively correlated with moral judgment.*

### 2.4. Perceived Quality, Moral Intensity and Moral Judgment

Perceived quality is depicted as the consumer’s estimation of a product’s overall excellence or preeminence [72]. It can be concluded that perceived quality is one of the most effective predictive factors of consumers’ purchase intention. For example, Suhud and Willson [73] selected perceived quality to forecast low-cost green car purchase intention and arrived at a conclusion that environmentally motivated vehicle purchase intention is positively and significantly impacted by perceived quality. Wasaya et al. [74] investigated 306 Pakistani consumers and found that perceived quality significantly predicted green purchase intention.

In addition to the quality factors of the product itself, context is also one of the key elements affecting consumers’ perception of quality [66]. If the overall consumption situation provides consumers with a positive and moral feeling, that contributes to promoting consumers’ recognition of product quality, thus positively affecting consumers’ perceptions of quality. Therefore, when the green product is more associated with environmental moral factors and the overall consumption situation is more positive, the consumer’s perception of its quality is enhanced, thus positively affecting the consumer’s moral intensity. Consequently, we suggest the hypothesis:

**Hypothesis** **5** **(H5).** *Perceived quality is positively correlated with moral intensity.*

In addition to factors such as product appearance, functional quality, and context, the environmental benefits that products represent may also be viewed by consumers during their assessment of overall perceived quality. Therefore, the environmental benefits of green products enhance consumers’ perceptions of their quality, and higher environmental benefits that bring more improvement to the environment positively affect the moral judgments of consumers. Given the aforementioned analysis, it can be supposed that higher perceived quality will promote consumers’ moral judgment. Consequently, we suggest the hypothesis:

**Hypothesis** **6** **(H6).** *Perceived quality is positively correlated with moral judgment.*

### 2.5. The Moderating Role of Products’ Green Degree

Xu et al. [75] developed a green-degree evaluation system for green products, to quantitatively evaluate the impact of different products on environmental performance, including material selection, long-term use, energy consumption, presence of hazardous substances, availability and rate of recycling, etc., to help governments choose more environmentally friendly products when purchasing.

Higher green degree of products means greater environmental benefit and lower environmental cost [76], and the relevant manufacturing costs, sales price, and even the perceived price by consumers increase accordingly. Meanwhile, higher perceived quality will be experienced by consumers. With improvement of their green degree, products are more helpful to the environment and ecology, allowing consumers to form more positive moral judgments [31]. Furthermore, an increased degree of greenness will enhance the moral intensity of consumers towards green products. Consequently, we suggest the hypotheses:

**Hypothesis** **7** **(H7).** *Products’ green degree positively moderates the relationship between perceived price and moral intensity.*

**Hypothesis** **8** **(H8).** *Products’ green degree positively moderates the relationship between perceived price and moral judgment.*

**Hypothesis** **9** **(H9).** *Products’ green degree positively moderates the relationship between perceived quality and moral intensity.*

**Hypothesis** **10** **(H10).** *Products’ green degree positively moderates the relationship between perceived quality and moral judgment.*

**Figure 1:**
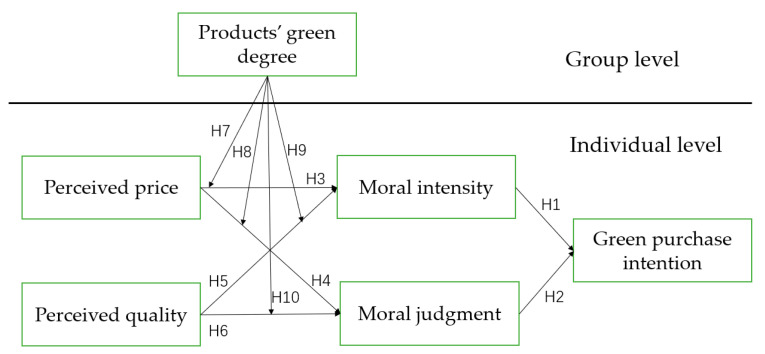
Research framework.

Based on the preceding work, the antecedents of the research framework were perceived quality and perceived price, and the outcome green purchase intention. Moral judgment and moral intensity were intermediate variables, and products’ green degree played a moderating role. Figure 1 depicts the research framework.

## 3. Methodology and Measurement

### 3.1. Measurement of Variables

This research used a questionnaire survey method to obtain data. In order to understand consumers’ intention to purchase environmentally friendly products, we selected green home appliances as representative of green products. Green home appliances are energy-efficient household appliances that have been designed to enhance energy efficiency of electrical items and reduce utility costs [77]. Zhang et al. [78] pointed out that household appliances such as refrigerators and washing machines are causes of high residential energy use and carbon emissions in China. The use of green home appliances can significantly contribute to the reduction of residential energy use and carbon emissions [78]. Green home appliances can reduce carbon emissions by 6.5 million tons annually, by saving roughly 10 billion kWh of power [79]. Research has shown that increasing the intention to buy green home appliances is critical to decreasing household usage of energy and associated carbon emissions [80]. Considering that green home appliances are widely consumed and used in general public and family life, in this study we selected green home appliances conducive to energy conservation and environmental protection as the typical green product for the associated questionnaire survey method.

Two components comprised the questionnaire used for this investigation. The first section included questions to gather information on the respondents’ gender, age, education, average monthly income, frequency of purchasing green home appliances in the past half year, average monthly expense on green home appliances. The second section was composed of questions related to the variables of this study, to obtain respondents’ perspectives on the suggested paradigm of this research; 30 questions were included. The relevant variables were measured using mature scales, including four items of perceived price adopted from Satriawan [71], four items of perceived quality adopted from Satriawan [71], three items of moral judgment adopted from Martinez and Jaeger [33], five items of moral intensity adopted from Lincoln and Holmes [60], three items of green purchase intention adopted from Sreen et al. [81], and eleven items of products’ green degree adopted from Tseng and Hung [82] (shown in Appendix A). These questions were devised using a five-point Likert scale, with 1 representing “strongly disagree” to 5 “strongly agree”.

### 3.2. The Sample and Data Collection

Through the Chinese internet survey platform Sojump (https://www.wjx.cn, accessed on 1 March 2022), we distributed structured questionnaires to customers. Each responder was chosen at random from the sample pool of the platform. Participants who had previously purchased and were acquainted with green home appliances were invited to take part in the survey, and responder was given a chance to win prizes in a lottery, such as online fitness courses or shopping coupons, as a token of gratitude. We distributed 398 questionnaires and 380 were retrieved within two months, 368 of which were valid, resulting in an effective response rate of 96.8 percent. Male participants comprised 45.9% of the total participants, and 55.8% of participants were in the age range 21 to 30, which accounted for the largest proportion of the total sample. Undergraduate students accounted for 28.6 percent of respondents, graduate students made up 28.2 percent, and the remainder were below undergraduate level. 48.9% of participants reported an average monthly income of between 3000 and 7000 RMB, with the remanent participants having average monthly incomes below (25.7%) or above (25.4%) this range. In the past half year, one to three purchases of green home appliances accounted for the heaviest proportion (56.68%), while 1~1000 yuan spent on green home appliances accounted for the most frequent reported monthly expense (42.12%).

## 4. Results

### 4.1. Reliability and Validity Analysis

The reliability of the scales was examined. All the scales, i.e., the perceived price scale (four items, α = 0.926), perceived quality (four items, α = 0.862), moral judgment (three items, α = 0.766), moral intensity (five items, α = 0.792), green purchase intention (three items, α = 0.869), and products’ green degree (eleven items, α = 0.856) surpassed the standard value of Cronbach’s alpha (0.7), indicating the scales’ high internal consistency.

This research examined the validity by three primary approaches, using construct validity, content validity, and criterion validity. The construct validity of the scale was analyzed using convergent validity and discriminant validity (see Table 1 and Table 2). As stated by Fornell and Larcker [83], when the square root of the average variance extracted (AVE) for each construct surpasses the corresponding correlations between that and any other construct, discriminant validity exists. The results showed that the minimum square root of AVE (0.654) exceeded the maximum correlation (0.518), so we accepted the discriminant validity. Additionally, AVEs of the six constructs were close to 0.5 or more than 0.5, while CRs of the six constructs were all above 0.7, and the other fit indexes all conformed to the standard values (GFI > 0.9, NFI > 0.9, AGFI > 0.8, RMR < 0.05), demonstrating that there was convergent validity. The existing scales, which have been empirically confirmed and shown to be acceptable, were utilized to establish content validity. Criterion validity was investigated using correlation analysis, as demonstrated in Table 2, demonstrating that the constructs performed credibly.

### 4.2. Null Model Analysis

The null model without considering any independent variables at two levels was employed to test the effect between groups. We calculated the intra-class correlation coefficient $\mathrm{ICC}=\frac{\tau_{00}}{\tau_{00}+\sigma^{2}}=0.085$ (see Table 3), indicating that 8.5% of the total variation of the subjects’ green purchase intention came from the between-group variation. According to Cohen [84], there is a moderate within-group correlation when 0.059 < ICC < 0.138, which indicates that there were considerable distinctions in green purchase intention within different groups. As a consequence, it was necessary to consider the hierarchical nested structure of the data rather than a general regression analysis model, thereby the hierarchical linear model was used as the analysis tool.

### 4.3. Cross-Hierarchy Analysis Results

To test the hypotheses proposed in this paper, we analyzed the data with HLM6.0 software utilizing the hierarchical linear model. In the analysis process, we took as control variables gender, age, education, average monthly income, times of purchasing green home appliances in the past half year, average monthly expense on green home appliances, to eliminate the influence of these individual-level variables.

Table 4 displays the findings of the correlation analysis: (1) Model 1 depicts the connection between moral intensity and green purchase intention, and the analysis proves that moral intensity had a significant positive impact on green purchase intention (γ = 0.324, *p* < 0.01); H1 is proved. This means that the moral intensity of consumers is triggered if certain consumption behaviors can bring benefit or harm to the environment or others. This result echoes previous research, which showed that consumers with higher moral intensity had a lower intention to purchase pirated software [32] and a higher intention to purchase organic products [57]. Therefore, moral intensity is an important consideration when consumers face ethical problems and think about how to make decisions [30]. (2) Model 2 depicts the connection between moral judgment and green purchase intention, and the analysis proves that moral judgment had a significant positive impact on green purchase intention (γ = 0.428, *p* < 0.001); H2 is proved. This indicates that when consumers make decisions about their consumption behavior, they judge whether purchasing green products is moral or immoral according to their own moral standards. Generally speaking, green products have positive effects on the overall environment, so they are associated with more positive moral factors than non-green products. This result is consistent with previous research, which found that the greater a person’s moral judgment, the less likely he is to acquire pirated software [32,62,63] or counterfeit fashion products [64]. (3) Model 3 depicts the connection between perceived price and moral intensity, and the analysis proves that perceived price had a significant positive impact on moral intensity (γ = 0.206, *p* < 0.01); H3 is proved. (4) Model 5 depicts the connection between perceived price and moral judgment, and the analysis proves that perceived price had a significant positive impact on moral judgment (γ = 0.213, *p* < 0.01); H4 is proved. (5) Model 3 depicts the connection between perceived quality and moral intensity, and the analysis proves that perceived quality had a significant positive impact on moral intensity (γ = 0.205, *p* < 0.01); H5 is proved. (6) Model 5 depicts the connection between perceived quality and moral judgment, and the analysis proves that perceived quality had a significant positive impact on moral judgment (γ = 0.227, *p* < 0.001); H6 is proved. The outcomes of (3)–(6) imply that the moral intensity and moral judgment of a consumer’s perception or behavior are affected by perceived price and perceived quality. Generally speaking, the perceived price and overall value of green products are higher than those of non-green products, bringing consumers more positive moral feelings and higher perceived price impressions in the long run. Therefore, when the perceived price of green products is higher, consumers perceive more obvious overall benefits and positive moral feelings about the product, enhancing their moral intensity and moral judgment. In addition, when consumers consider whether there is a viable alternative product, the moral intensity of the purchase is affected. If fungible general products and green products are similar in price and quality, consumers’ moral intensity may increase when they purchase goods, believing that it is better to buy environmentally friendly products. Similarly, consumers are more likely to make better moral judgment decisions if they perceive the quality and function of green products to be more beneficial to environmental protection.

The results of the moderation analysis are shown in Table 4: (7) Model 4 shows the relationship between products’ green degree × perceived price and moral intensity, and the analysis proves that the moderating effect of products’ green degree on the relationship between perceived price and moral intensity was not significant (γ = 0.263, *p* > 0.05); H7 is rejected. (8) Model 6 shows the relationship between products’ green degree × perceived price and moral judgment, and the analysis proves that products’ green degree significantly moderated the relationship between perceived price and moral judgment (γ = 0.336, *p* < 0.01); H8 is proved; (9) Model 4 shows the relationship between products’ green degree × perceived quality and moral intensity, and the analysis proves that that the moderating effect of products’ green degree on the relationship between perceived quality and moral intensity was not significant (γ = 0.167, *p* > 0.05); H9 is rejected; (10) Model 6 shows the relationship between products’ green degree × perceived quality and moral judgment, and the analysis proves that products’ green degree significantly moderated the relationship between perceived price and moral judgment (γ = 0.465, *p* < 0.001); H10 is proved. The findings of (7)–(10) suggest that products’ green degree did not moderate the relationships between perceived price or quality and moral intensity, contradicting the preceding inference assumptions. The main reason for this is that the level of moral intensity is primarily influenced by moral factors [31] rather than personal or product factors. As a result, although the perceived price, overall environmental benefits, and positive morality of green products are higher than those of non-green products, they cannot directly affect moral intensity. Furthermore, products’ green degree positively moderated the impact of perceived price on moral judgment, which is consistent with the previous inferences. Green products can provide greater environmental benefits, or generate less environmental cost than non-green products [76], often incurring increased associated production costs. Consequently, the higher a product’s green degree, the greater its value and benefits, thereby strengthening the influence of perceived price on consumers’ moral judgment. On the other hand, products’ green degree played a positive moderating role in the influence of perceived quality on moral judgment. When the perceived quality felt by consumers is greater, it indicates that the overall quality level of the product at the moment of purchase is excellent, which in turn bring consumers a positive feeling and affects their moral judgment. Compared with non-green products, green products can bring consumers a sense of the value of their environmental benefits. As a result, when a product’s green degree is higher, it can convey a more positive moral message, thereby enhancing the impact of perceived quality on consumers’ moral judgment.

### 4.4. Results Summary of the Hypothesis Test

The research findings are reported based on the data analysis. Table 5 displays the findings of this study’s research assumptions.

## 5. Discussion

### 5.1. Research Implications

The results have three significant implications for future research on green purchase intention. First, considering the lack of research on the factors affecting green purchase intention from a moral perspective, this study focuses on the impact of moral judgment and moral intensity on green purchase intention. This will be beneficial to enrich the research on consumers’ green purchase intentions, and to expand the applicability of ethical decision-making research in different contexts. Second, previous research primarily paid attention to the direct influence of value on green purchase intention [45,46,47], and scholars have rarely investigated the intermediate process of the respective impact of perceived quality and perceived price on green purchase intention. Therefore, this study further explores the impact of consumers’ perceived quality and price on moral judgment and moral intensity, providing a more complete framework to comprehend the influencing variables affecting consumers when purchasing green products. Third, this study adds to prior research by exploring the moderating role of products’ green degree in relation to green purchase intention. Specifically, the results offer empirical proof that, in the presence of a high degrees of product greenness, perceived price and perceived quality have a favorable influence on moral judgment, which ultimately encourages consumers to make green purchases.

### 5.2. Managerial Implications

This research has practical consequences for marketers developing marketing strategies. Enterprises could add moral factors and issues to their marketing advertisements, such as global warming, garbage pollution, biological extinction, etc., to trigger consumers’ moral judgment and moral intensity, thus increasing their green purchase intension. Perceived price and perceived quality affect consumers’ ethical decision-making processes. Therefore, in addition to enhancing the overall quality level and environmental benefit of green products through research and development, enterprises can also display more conspicuous environmentally friendly labels on product packaging, to improve the perceived price and perceived quality of green products. On the other hand, to enhance consumers’ faith and green purchase intention, enterprises should actively improve the green degree of their products, further striving for green-related certifications and awards while creating a green brand image. Enterprises should also attach significance to the findings that the frequency of purchasing green products had a significant negative impact on moral judgment; to reduce this impact, marketing strategy makers could therefore strengthen the positive emotional connection between brands and consumers.

### 5.3. Limitations and Future Research

There remain some deficiencies to be resolved. First, due to the limitation of resources and time, this study adopted cross-sectional analysis. Subsequent research can use longitudinal analysis and conduct long-term collection of consumer data to assess effectively the changes in relationships between variables, in order to reduce error arising from common-method variance. Second, the generality of our findings remains to be proven. Selecting green home appliances as the research object, the internal logical relationships between perceived quality, perceived price, moral judgment, moral intensity, and green purchase intention were empirically tested in China; future research can collect further larger samples of green products from diverse countries to prove the applicability of the conclusions. Third, although behavioral intention is one of the direct determinants of behavior, many factors can prevent the implementation of intended behavior. Future studies may explore the relationship between customers’ green purchase intentions and actions. Scholars may also investigate other intermediate factors, including subjective norms and different moderating factors such as brand green image, to increase the depth and scope of studies of how perceived quality and price affect customers’ inclination to buy green products.

## 6. Conclusions

Environmental protection issues are closely related to moral factors, but little research has explored the factors affecting green purchase intention from the aspect of morality. Based on a perspective of ethical decision making, this study investigated the impacts of perceived quality and perceived price on moral intensity and moral judgment through the moderation of products’ green degree, as well as the impacts of moral intensity and moral judgment on green purchase intention. Results revealed that moral intensity and moral judgment both significantly boosted green purchase intention. Perceived quality and perceived price both positively affected moral judgment and moral intensity. Products’ green degree positively moderated the relationship between perceived price and moral judgment as well as the relationship between perceived quality and moral judgment.

This study contributes not just to the advancement of research on consumers’ green purchase intention, but also to the expanding understanding of ethical decision making in a variety of circumstances. Moreover, it provides a supplement to the current literature by exploring the moderating influence of products’ green degree on green purchase intention. The findings might serve as a guide for marketers to prioritize perceived quality and price, concentrate on moral judgment and moral intensity, and pay attention to how products’ green degree affects green purchasing.

## Figures and Tables

**Table 1:** Convergent validity analysis.

Constructs	Cronbach’s α	CR	AVE	GFI	NFI	AGFI	RMR
Perceived price	0.926	0.915	0.638	0.964	0.972	0.905	0.022
Perceived quality	0.862	0.844	0.594	0.955	0.938	0.862	0.025
Moral judgment	0.766	0.805	0.490	0.958	0.918	0.905	0.035
Moral intensity	0.792	0.796	0.448	0.979	0.956	0.959	0.019
Green purchase intention	0.869	0.708	0.496	0.976	0.928	0.822	0.021
Products’ green degree	0.856	0.852	0.446	0.945	0.913	0.898	0.028

**Table 2:** Correlation analysis and discriminant validity.

Variables	Mean	SD	1.	2.	3.	4.	5.	6.
1.Perceived price	3.548	0.782	0.799 ^a^					
2.Perceived quality	3.985	0.552	0.458 ***	0.771 ^a^				
3.Moral judgment	3.983	0.446	0.420 ***	0.319 ***	0.700 ^a^			
4.Moral intensity	3.995	0.452	0.316 ***	0.293 ***	0.435 ***	0.654 ^a^		
5.Green purchase intention	3.988	0.382	0.279 ***	0.356 ***	0.310 ***	0.364 ***	0.704 ^a^	
6.Products’ green degree	3.912	0.465	0.518 ***	0.506 ***	0.426 ***	0.344 ***	0.305 ***	0.668 ^a^

Notes: *** *p* < 0.001 (two-tailed), N = 368; diagonal value ^a^ is the square root of AVE for each construct.

**Table 3:** The null model statistics of green purchase intention.

Within-Group Variation ($\sigma^{2}$)	Between-Group Variation ($\tau_{00}$)	*p*-Value	ICC
0.195	0.018	0.022	0.085

**Table 4:** Cross-hierarchy analysis results.

Models		Model 1	Model 2	Model 3	Model 4	Model 5	Model 6
	Dependent Variables	GPI		MI		MJ	
Independent Variables							
**Individual level**							
Intercept		3.472 ***	3.663 ***	4.196 ***	4.204 ***	4.056 ***	4.027 ***
Gender		0.125	0.064	−0.010	−0.006	0.021	0.028
Age		0.038	0.019	−0.009	−0.008	0.003	0.001
Education		0.044	0.033	−0.085	−0.096 *	−0.015	−0.005
Average monthly income		0.035	0.020	0.028	−0.008	0.037	0.038
Times of purchasing green home appliances in the past half year		−0.040	0.009	−0.035	−0.007	−0.093	−0.110 *
Average monthly expense on green home appliances		0.012	−0.013	0.031	0.035	0.027	0.022
PP				0.206 **		0.213 **	
PQ				0.205 **		0.227 ***	
MI		0.324 **					
MJ			0.428 ***				
**Group level**							
PGD × PP					0.263		0.336 **
PGD × PQ					0.167		0.465 ***
σ^2^		0.122	0.116	0.119	0.126	0.097	0.115
Deviance		287.052	275.608	268.195	276.904	273.854	289.657

Notes: * *p* <0.05, ** *p* < 0.01, *** *p* < 0.001 (two tailed); PP = perceived price, PQ = perceived quality, PGD = products’ green degree, GPI = green purchase intention, MI = moral intensity, MJ = moral judgment.

**Table 5:** Results summary for each hypothesis.

Hypothesis	Content	Result
H1	Moral intensity is positively correlated with green purchase intention.	Supported
H2	Moral judgment is positively correlated with green purchase intention.	Supported
H3	Perceived price is positively correlated with moral intensity.	Supported
H4	Perceived price is positively correlated with moral judgment.	Supported
H5	Perceived quality is positively correlated with moral intensity.	Supported
H6	Perceived quality is positively correlated with moral judgment.	Supported
H7	Products’ green degree positively moderates the relationship between perceived price and moral intensity.	Not supported
H8	Products’ green degree positively moderates the relationship between perceived price and moral judgment.	Supported
H9	Products’ green degree positively moderates the relationship between perceived quality and moral intensity.	Not supported
H10	Products’ green degree positively moderates the relationship between perceived quality and moral judgment.	Supported

## Data Availability

The data that support the findings of this study are available from the corresponding author, upon reasonable request.

## Appendix A

**Table A1:** Details of the scales.

Constructs	Numbers	Content	Resources
Perceived price	PP1	Green home appliances have economical prices compared to non-green home appliances.	Satriawan [71]
	PP2	Green home appliances’ prices offered align with the qualities obtained.	
	PP3	Green home appliances have affordable prices.	
	PP4	The prices of the green home appliances correspond with my purchasing power.	
Perceived quality	PQ1	The performances of green home appliances are very good.	Satriawan [71]
	PQ2	Green home appliances have long use life.	
	PQ3	Green home appliances have specifications that fit my desires.	
	PQ4	Green home appliances have complete features.	
Moral judgment	MJ1	I consider the purchase of green home appliances to be morally acceptable.	Martinez and Jaeger [33]
	MJ2	The act of buying green home appliances rather than non-green home appliances is right.	
	MJ3	It is morally right to buy green home appliances.	
Moral intensity	MI1	The possible harm resulting from the purchase decision of green home appliances is minor.	Lincoln and Holmes [60]
	MI2	Any negative consequences of green home appliance purchase decision are likely to occur after a long time.	
	MI 3	Most consumers would consider green home appliance purchase decision to be appropriate.	
	MI 4	The green home appliance purchase decision would negatively affect people outside of my group.	
	MI 5	The chances of any negative consequences not likely to occur as a result of the green home appliance purchase decision.	
Green purchase intention	GPI1	I intend to buy green home appliances.	Sreen et al. [81]
	GPI2	I plan to purchase green home appliances.	
	GPI3	I will buy green home appliances in my next purchase.	
Products’ green degree	PGD1	Green home appliances label the product ingredients clearly.	Tseng and Hung [82]
	PGD2	Green home appliances have eco-labels.	
	PGD3	Green home appliances also have attractive appearances.	
	PGD4	The operation of green home appliances is user-friendly.	
	PGD5	Green home appliances are made from non-polluting materials.	
	PGD6	Green home appliances have a high recyclability ratio.	
	PGD7	Green home appliances have high energy conservation rates.	
	PGD8	Green home appliances use recycled packaging materials.	
	PGD9	Green home appliances have good functional performance.	
	PGD10	The design and operating characteristics of green appliances are suitable.	
	PGD11	Green home appliances have good durability.	
